# Surrogate-based optimization with adaptive sampling for microfluidic concentration gradient generator design

**DOI:** 10.1039/d0ra01586e

**Published:** 2020-04-06

**Authors:** Haizhou Yang, Seong Hyeon Hong, Rei ZhG, Yi Wang

**Affiliations:** Department of Mechanical Engineering, University of South Carolina Columbia SC 29208 USA yiwang@cec.sc.edu; Department of Traffic Information and Control Engineering, Tongji University Shanghai 200092 P. R. China

## Abstract

This paper presents a surrogate-based optimization (SBO) method with adaptive sampling for designing microfluidic concentration gradient generators (μCGGs) to meet prescribed concentration gradients (CGs). An efficient physics-based component model (PBCM) is used to generate data for Kriging-based surrogate model construction. In a comparative analysis, various combinations of regression and correlation models in Kriging, and different adaptive sampling (infill) techniques are inspected to enhance model accuracy and optimization efficiency. The results show that the first-order polynomial regression and the Gaussian correlation models together form the most accurate model, and the lower bound (LB) infill strategy in general allows the most efficient global optimum search. The CGs generated by optimum designs match very well with prescribed CGs, and the discrepancy is less than 12% even with an inherent limitation of the μCGG. It is also found that SBO with adaptive sampling enables much more efficient and accurate design than random sampling-based surrogate modeling and optimization, and is more robust than the gradient-based optimization for searching the global optimum.

## Abstract 2

Surrogate-based optimization (SBO) with adaptive sampling method is established for microfluidic concentration gradient generators (μCGGs) design.

## Graphical abstract

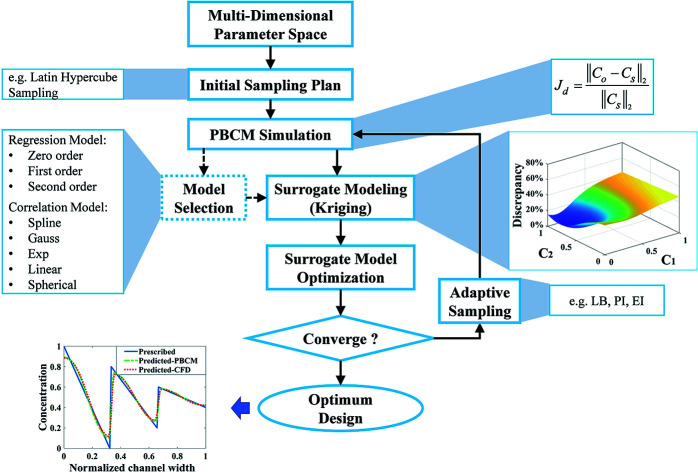

## 1 Introduction

Formation of complex concentration gradients (CGs) of biomolecules plays an important role in biological processes,^1^ such as immune response, wound healing, embryogenesis, cancer metastasis, and others. One active research area is to generate and maintain concentration gradients, such as linear, parabolic, exponential, sawtooth, and hybrid profiles^2–4^ using microfluidic devices. In contrast to their counterparts at the macroscale, the microfluidic concentration gradient generator (μCGG) features several unique merits, including short transportation time, fast analysis speed, simple operation, precise manipulation of locations and quantities of biomolecule delivery, and excellent physiological capability to cellular assays at spatiotemporal scales.^5–8^ Therefore, a variety of μCGGs are designed, microfabricated, and demonstrated in the field of cell biology and biochemistry, including tree-shaped, altered tree-shaped, Y-shaped, pressure-balanced, incomplete mixing-based, and membrane μCGGs.^6,9^ The tree-shaped network is one of the earliest μCGG designs, which successively splits, mixes, and recombines biologically relevant chemical solution to form digitalized CGs across channel widths.^10,11^ In order to generate more complex CGs with higher resolutions, the number of stages of tree-shaped μCGG needs to be increased, which however may be more prone to clogging or leakage.^6,12^ Therefore, an altered tree-shaped device was developed, which is able to reduce the number of stages of the tree-shaped network and simplify the structure by delicately designed splitting-and-combining patterns.^13^ Moreover, a Y-shaped generator is designed to simplify the structure compared to conventional and altered tree-shaped networks by reducing the mixing channel length.^9^ In contrast to these complete mixing-based μCGGs, μCGGs utilizing partial mixing were also proposed by our coauthor that manipulates species transport within microchannels and juxtaposes constituent CGs to form complex ones, leading to simple network topology and salient device reliability.^12,14^ μCGGs are also proposed to separate the flow by a porous membrane and generate CGs by only permitting specific molecules to pass through, and hence, yielding shear free CGG.^15^

Research efforts above mostly focused on demonstrating μCGGs that were fabricated with known operating parameters, such as inlet concentrations and pressures/flow rates. In general, determining these design parameters is challenging, a trial-and-error process entailing iterative modeling, simulation, and experiments under the guidance of prior experiences. Therefore, a component model and systematic simulation-based μCGG design method^12,14^ was previously proposed for designing partial mixing-based μCGGs. It proceeds iteratively within the design space to search for the combination of operating parameters yielding the best agreement with the prescribed CGs. However, the process was performed manually, and could be further improved by automated optimization. Friedrich *et al.*^16^ utilized a μCGG consisting of a single microfluidic channel and an obliquely angled groove, which is designed by optimization using CFD simulations, to generate a prescribed CG, such as linear and exponential. An efficient μCGGs design automation method based on physics-based models and simulation to rapidly determine operating parameters that accurately generate prescribed CGs is indeed scarce and strongly needed. In this context, we propose a surrogate-based optimization (SBO) with adaptive sampling framework to address such a challenge. The key elements of our proposed method include: first, optimization is undertaken on the surrogate model and searches within the design space for optimal parameters that can generate CGs matching the prescribed ones. Surrogate models, also known as response surface models and metamodels are used to approximate the behavior of physics-based models through direct mapping between input–output data pairs produced by the latter, and is more computationally efficient to evaluate. Therefore, they are widely used to minimize the number of evaluations by physics-based computer simulation, such as the computational fluid dynamics (CFD) or the computational structural dynamics (CSD)^17,18^ for accelerated optimization and design process. It is well known that high-fidelity, physics-based simulation can be computationally prohibitive for optimization in high-dimensional design parameter space.^19,20^ The surrogate model, constructed by a small number of selected physics-based simulations, enables a cost-effective and rapid exploration of the design space, thereby making it feasible and robust to locate the global optimum.^17,21,22^ Second, an adaptive sampling and infill strategy is utilized to determine new sample points at the most important but under-explored regions for the next round of physics-based simulation to progressively improve surrogate model accuracy, especially near the region of the global optimum by analyzing its underlying response surface. The infill is undertaken with respect to a criterion that balances between exploitation and exploration.^23^ Last, a physics-based, component modeling (PBCM) approach we developed previously to analyze species transport in μCGGs^12^ will be employed as the main engine to generate simulation data for surrogate model construction and SBO. Because of its closed-form nature, the PBCM simulation can typically run orders of magnitude faster than high-fidelity CFD simulation, and therefore, is used to generate simulation data for surrogate modeling. In the previous work, the PBCM method was verified by both CFD simulation^12^ and experiments^14^ for a variety of CGs, including linear, saw-tooth, and bell shapes, and proven valid for a variety of μCGGs.^12^

In contrast to existing efforts of μCGG modeling and design, this paper presents several novelties. First, to the best of our knowledge, it is an initial effort to establish SBO with adaptive sampling/infill method for μCGG design. Second, a comparative analysis is carried out to thoroughly investigate the effects of various combinations of correlation functions, regression functions, and infill strategies on surrogate model accuracy and SBO convergence for μCGG design. Last, a new formulation for SBO of μCGGs is proposed to avoid the backflow issue, that is, liquid solution unexpectedly exits through inlets of the μCGG network due to overly large difference of the pressure head among inlets. In this formulation, instead of the inlet pressures, the pressure differences between branch points within the μCGG network are used as design variables, which facilitates surrogate modeling and adaptive sampling (see Section 3 for details). Note that the new formulation can potentially be extended to microfluidic electrokinetic flow driven by the electric field.^24,25^

This paper is organized as follows. The methodology of the SBO method for μCGGs is introduced in section 2, which describes the PBCM, surrogate modeling, and different infill strategies. Section 3 elucidates the problem formulation and case studies. In Section 4, the results of SBO with adaptive sampling for prescribed CGs of various profiles are discussed. Finally, this paper concludes with a summary in Section 5.

## 2 Methodology

**Fig. 1:**
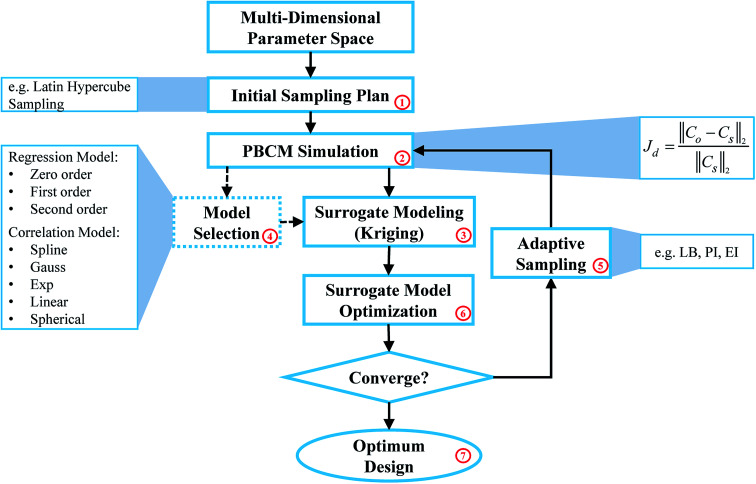
Flowchart of the SBO with adaptive sampling for μCGG.

Fig. 1 illustrates the SBO process with adaptive sampling,^19,23,26,27^ specifically for designing inlet operating parameters of μCGGs that allow generating user-desired/prescribed CGs. It includes initial sampling, model selection, surrogate modeling, surrogate model optimization, adaptive sampling (or infill), and iterative surrogate model update to gradually identify the global optimum parameters within the design space. The detailed procedure is given as follows: first, latin hypercube sampling (LHS) (block labeled ‘1’ in Fig. 1), one kind of the one-shot space-filling techniques for the design of experiments (DoE), is used to generate initial samples in the multi-dimensional design space,^28,29^ which includes chemical concentrations at the inlet reservoirs and pressures (or flow rates). Second, the aforementioned physics-based component models (PBCM)^12^(labeled ‘2’ in Fig. 1) representing the designated μCGG network is then simulated to predict corresponding CGs at each sample obtained in the previous step. The discrepancy *J*_d_ between the generated CG *C*_o_ at the sampled point and the user-prescribed CG *C*_s_, *i.e.*, the Normalized Root Mean Squared Error (NRMSE)^30^ is used as the output of the surrogate model. Next, the existing sampled points and their corresponding discrepancies *J*_d_s relative to the user-prescribed CG are utilized as the input-out data pairs to construct the surrogate model (labeled ‘3’ in Fig. 1). Despite a variety of surrogate model techniques available to establish the input–output mapping relationship,^23^ the Kriging interpolation method that is comprised of a trend regression model and a correlation model is adopted in this work. Because of multiple choices of the regression model and the correlation model, the best combination of them needs to be selected and will be used for subsequent infill and SBO. Therefore, a model selection process (labeled ‘4’ in Fig. 1) will be executed using the initial sampling data during the first iteration. That is, the data of initial sampling is divided into two subsets, and the first subset is used to construct the surrogate model, while the second to evaluate its accuracy.

Since the surrogate model is an approximation of the physics-based model, an adaptive sampling technique (also known as an infill) (labeled ‘5’ in Fig. 1) will be incorporated into SBO, which during each iteration will add a new sampled point and its corresponding discrepancy *J*_d_ computed by PBCM (labeled ‘2’) into the data set to update the surrogate model (labeled ‘3’) for enhanced accuracy. Essentially infill is a sub-optimization process to identify a new sample within the design space that minimizes or maximizes a specific infill criterion, and hence, providing more information than randomly selected samples for SBO. In addition, the surrogate model is very computationally efficient, and each evaluation only costs milli- to centi-second. As a result, it can be used to find the global optimum, *e.g.*, using the genetic algorithm that entails a large number of model evaluations. The infill, PBCM simulation, and optimization will be repeated until the minimum of the surrogate model (labeled ‘6’ in Fig. 1) converges with respect to a predefined tolerance or the maximum number of iterations defined by the user is reached. Once converged, the optimum design (labeled ‘7’ in Fig. 1), selected from the minimum of the surrogate model and all existing samples in the last iteration, will be supplied to PBCM and CFD simulation to predict corresponding CGs, which then will be compared with prescribed CGs to verify SBO-based design of μCGGs. The detailed verification process is elucidated in Section 4.

### 2.1 Microfluidic concentration gradient generators and physics-based component model

**Fig. 2:**
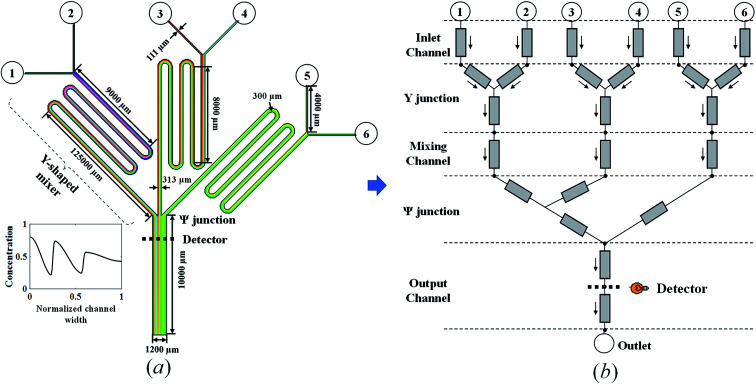
(a) Schematic and geometric parameters and (b) physics-based component model of the triple-Y μCGG.

The proposed SBO with adaptive sampling is performed on a triple-Y μCGG that was reported in our previous paper.^12,14^ It is comprised of three Y-shaped mixers combined through one Ψ-shaped junction that is then followed by a main output microchannel as shown in Fig. 2a, that is, in total there are six inlets and one outlet, respectively, located at the top and the bottom. In each Y-shaped mixer, two streams containing chemicals of different concentrations enter the μCGG *via* the two inlets, and then merge together and diffuse transversely within the mixing channel following the Y-junction. At the end of the mixing channel, a monotonically increasing or decreasing linear CG is generated. Subsequently, constituent CGs emanating from all the three Y-shaped mixers are concatenated along the width direction in the Ψ-shaped junction to form an even more complex CG at the entrance of the main output channel. Likewise, the chemicals carried by the three streams will also diffuse within the main output channel, and the extent of mixing depends on the location relative to the entrance. Both the chemical concentrations at the inlets and the pressure (or equivalently the flow rates) can be used to tune precisely the generated CGs. For example, a large flow rate driven by a large pressure head applied to the inlet will reduce the residence time of the chemical and inter-stream diffusion within the microchannels, resulting in a sharp gradient of the chemical concentration. On the other hand, a small flow rate and pressure head leads to milder CGs. In addition, unequal pressure or flow rates among the three Y-shaped mixers will also give rise to different widths of the constituent CGs in the concatenated one.

Although high-fidelity CFD can be used to simulate the μCGG above to produce data for surrogate modeling, the physics-based component modeling (PBCM) method verified by both CFD simulations^12^ and experiments^14^ is adopted instead in this work. In our prior research, PBCM demonstrated excellent speedup without appreciably compromising simulation accuracy relative to CFD. In the PBCM method, a μCGG network of complex topology, such as the one in Fig. 2a, can be decomposed into a set of constituent components, including microchannels (straight or curved), Y-junctions, inlet reservoirs, and outlet reservoirs as shown in Fig. 2b. The simple geometries of these constituent components render possible the analytical solution of their underlying species transport equation. The component models are then connected in correspondence to the desired μCGG topology to form a network model that can be simulated at a fast speed because of its analytical, closed-form nature.

PBCM considers the fluid flow and the species transport separately within each constituent component above and is only applicable to μCGGs. Since the full set of the models were reported previously,^12,14^ the important ones for the microchannel and the Y-junction are described here briefly for the sake of completeness of the paper. The microchannel is used for mixing and diffusion of chemicals along the channel width to form desired CGs. The fluid flow within the microchannel is modeled using the electric analogy and its hydrodynamic resistance is given in our previous work.^12^ To model the species transport, two assumptions are taken, that is, the channel is flat with a large aspect ratio and long. With a flat channel, the effect on the chemical transportation due to nonuniform velocity distribution along the channel cross-section is negligible and the convection term in the transport equation can be approximated by the cross-sectionally averaged velocity. Within a long channel, the axial diffusion is also negligible.^12^ The simplification allows analytical solution to the convection–diffusion equation, in which the chemical concentration is represented by a Fourier series, and the relationship of the Fourier coefficients (*d*_*n*_) between the inlet and the outlet is given by1
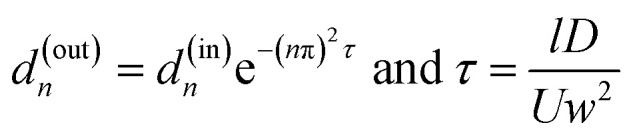
where *l* is the channel length, *D* is the molecular diffusivity of the chemical, *w* is the channel width and *U* is the average flow velocity.

For the Y-junction, two streams enter from the inlets, and are combined as a single stream exiting through the outlet. The flow resistance between the inlets and the outlet of the Y-shaped junction is assumed zero, that is, it is treated as a point-wise component without the physical size. The relationship between Fourier coefficients *d*^(in)^_*n*_ and *d*^(out)^_*n*_ of the concentration profile at the inlets and the outlet is2
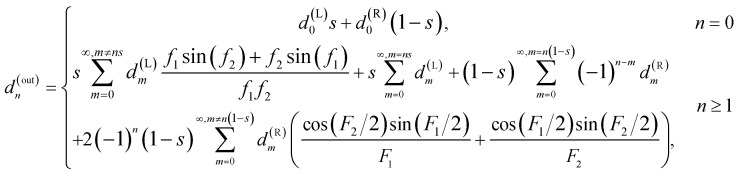
where L, R and out denote the left inlet, right inlet, and outlet, respectively; *s* = *q*^(L)^/(*q*^(L)^ + *q*^(R)^) denotes the flow ratio of the left stream to the right stream at the Y-junction, and also the normalized position of the stream interface; *q* is the flow rate. *f*_1_ = (*m* − *ns*)π, *f*_2_ = (*m* + *ns*)π, *F*_1_ = (*m* + *n* − *ns*)π and *F*_2_ = (*m* + *n* + *ns*)π. A Ψ-shaped junction consisting of three inlets and one outlet can be treated as a cascade concatenation of two Y-shaped junctions as shown in Fig. 2b, and the Fourier coefficients are obtained by solving eqn (2) twice. That is, the Fourier coefficients at the outlet of the first Y-shaped junction is supplied to the left inlet of the second Y-shaped junction.

All the PBCMs above in this work are developed in MATLAB (https://www.mathworks.com), and the simulation is carried out in two serial steps. First, the pressure and the flow distribution within the μCGG network is simulated following the Kirchhoff's law given the boundary conditions, *i.e.*, the pressure and/or flow rate specified at the inlet and outlet reservoirs. Next, the Fourier coefficients of the concentration profiles are calculated along the flow direction determined in the previous step, and the calculation is initiated from inlet reservoirs where constant concentrations of the chemical are specified as the design variables in SBO. The coefficients {*d*^(out)^_*n*_}^*j*^ at the outlet of the *j*^th^ component are computed using those at its inlet(s), and then assigned to those at the inlet of the component immediately downstream. It should be noted that PBCM above is applicable to both the partial mixing- and the complete mixing-based μCGG,^12^ while in this paper only demonstrated for the former that involves species transport along the width of each component and is more challenging to design.^10^

### 2.2 Surrogate modeling: universal Kriging

The Kriging interpolation method first proposed by Krige and Sacks is mainly used to predict the unknown response based on existing samples by minimizing prediction's mean squared error (MSE).^21^ Universal Kriging is one of the kriging methods, and comprised of a polynomial regression model, *f*^*T*^(*x*)*β* to represent the global trend of the sampled data, and a correlation model, *Z*(*x*) to capture the distance from the data points to the regression surface.^19,31^ Mathematically the universal Kriging interpolation reads

3 *y*(*x*) = *f*^*T*^(*x*)*β* + *Z*(*x*), *x* ∈ *R*^*k*^

where *k* is the dimension of input variables; *f*^*T*^(*x*) = [*f*_0_(*x*), *f*_1_(*x*), …, *f*_*n*−1_(*x*)]^*T*^ is a set of basis functions of regression, *e.g.*, zero, first, and second-order polynomial terms; *β* is the vector of regression coefficients; *n* is the number of the regression basis functions. The correlation model *Z*(*x*) represents a random stochastic process with zero mean and *σ*^2^ variance, and the covariance and the correlation matrix for the process are defined, respectively, in eqn (4) and (5)

4 Cov(*Z*, *Z*) = *σ*^2^*Ψ*

5
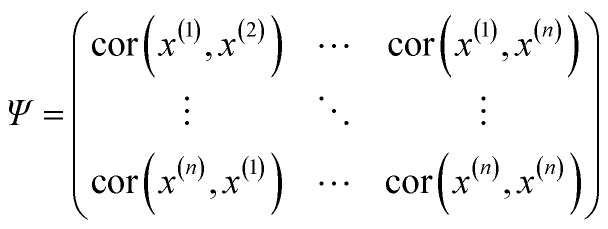
where ‘cor’ denotes a correlation function that depends on the Euclidean distance between two data points. Widely used correlation functions include Gaussian, spline, exponential, linear, and spherical.

### 2.3 Adaptive sampling and infill

Adaptive sampling and infill, is a key technique that exploits response surface information of the existing surrogate model and adds new samples and information at critical regions within the design space to further refine the surrogate model for optimization.^32^ Through a discreet selection of infill points, accurate surrogate models can be constructed with a small number of samples.^28^ Normally the infill process is repeated until stopping criteria are satisfied, such as the number of maximum iterations and error tolerance. As shown in Fig. 1, the infill is embedded in the optimization loop, the choice of infill techniques and criteria is critical for SBO performance. In this work, three different infill techniques: statistical lower-bound (LB), probability of improvement (PI), and expected improvement (EI) are applied, evaluated, and compared. The statistical lower-bound (LB) is defined as:

6 LB(*x*) = *ŷ*(*x*) − *Aŝ*(*x*)

where *ŷ* and *ŝ* are the prediction and MSE of the surrogate model at the input variable *x*, respectively. *A* is a constant that balances between the exploitation and exploration^23^ for sample selection, and in this paper, an empirical value of *A* = 2 is accepted. Exploitation uses the information of the optimum in the current iteration, and select infill points close to it. It is well suited for the scenario when the global optimum is relatively easy to find. Exploration focuses on the regions that are unknown or under-sampled, and then adds infill points where the surrogate model exhibits the large MSE, and hence, is more suitable for complex problems where multiple optimums appear in the response surface, such as the multi-modal response surface. In this paper, the infill point is obtained by finding the sample location that minimizes the statistical lower-bound.

Probability of improvement (PI) selects an infill point that leads to a maximum probability of an improvement, *I* = *y*_min_ − *ŷ*(*x*),^33^ where *y*_min_ is the minimum observation of existing samplings. PI is expressed as an error function as shown in eqn (7), and maximizing it yields the infill point7
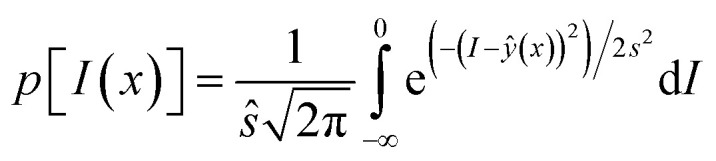


A quantity of expected improvement (EI) within the design space can also be defined to balance the need of exploitation and exploration to improve the surrogate model.^34^ The infill is essentially to maximize the amount of improvement we expect.^23^ The equation of EI is shown in eqn (8).8
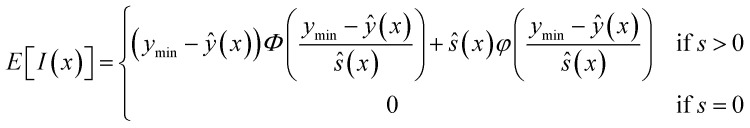
where *Φ*(*x*) and *φ*(*x*) are the cumulative distribution function and the probability density function, respectively. This equation represents the area enclosed by the Gaussian distribution below the minimum of surrogate model found at the current iteration.^19^ Similar to PI, the infill point is attained by maximizing the EI in eqn (8).

### 2.4 Genetic algorithm

For SBO with adaptive sampling, global optimization needs to be performed for sample infill and search for the minimum of *J*_d_ (Min. *J*_d_) using the surrogate model. Despite various global optimization methods, the genetic algorithm (GA)^35^ is selected for use. GA evolves an initial population of random gene sequences, through many generations, and toward a final population of “fit” gene sequences that demonstrate optimal performance on a fitness function used to assess the performance of a given gene sequence. There are three basic genetic operations, reproduction, cross over, and mutation,^36^ which need to be undertaken to generate the next generation with consideration of both exploration and exploitation. The GA process will be terminated by evaluating certain stop criteria, *e.g.*, meeting the desired tolerance in the fitness value or reaching the maximum number of generations.

## 3 Problem formulation and case studies

**Fig. 3:**
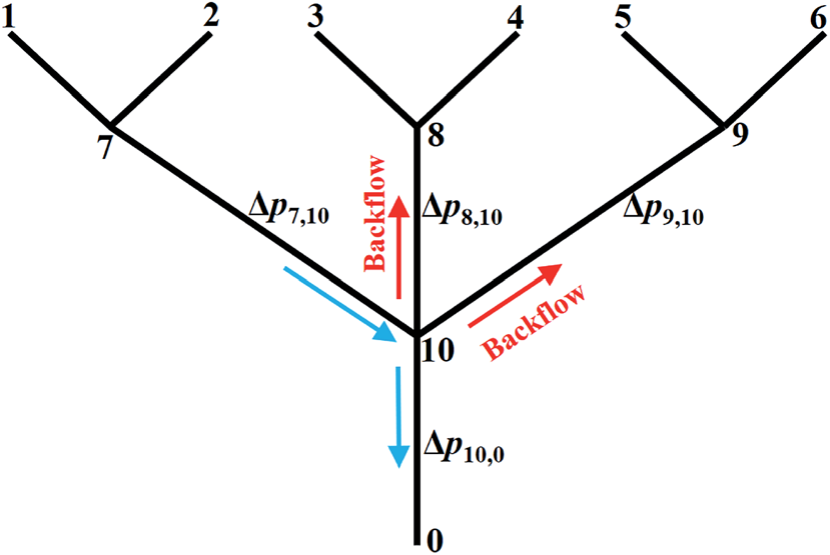
Illustration of the backflow issue and reformulation of the design problem to use the pressure difference as the design variables rather than the inlet pressure for the triple-Y μCGG.

In this section, the problem of μCGG design will be formulated, including the design variables and the cost function used in SBO, and case studies used to verify the proposed method will also be described. For the μCGG presented in this paper, all channels have a depth of *h* = 60 μm with the aspect ratio of 5–20, and other geometric parameters of the μCGG are given in Fig. 2a.^12,14^ PBS buffer with a viscosity of 0.001 kg m^−1^ s^−1^, and the chemical with diffusivity 1 × 10^−10^ m^2^ s^−1^ is used. The first step of our SBO design formulation is to prescribe a desired CG *C*_s_, such as the linear, sawtooth, trapezoidal, and others. The cost function is then defined as the discrepancy *J*_d_ between the CG created by the candidate design *C*_o_ at the detector location and the prescribed one *C*_s_. It seems that the inlet parameters of the triple-Y μCGG should be used as the design variables to minimize *J*_d_, such as inlet concentrations *C*_*i*_ and pressures *p*_*i*_ (or equivalently the flow rate *q*_*i*_) at the inlet, where *i* denotes the *i*^th^ inlet. However, as shown in Fig. 3, when the pressure at junction 7 is greatly higher than those at junction 8 or (and) 9 and the outlet 0, the pressure at junction 10 may also be higher than that at Junction 8 or (and) 9. Thus a fraction of fluid from the first Y-shaped mixer will be diverted towards the second and the third Y-shaped mixer, and unexpectedly exit through inlet 3, 4, 5 and 6, *viz*., backflow, although the inlets are originally intended for inflow. To eliminate such an issue, the pressure difference between junctions in each Y-shaped mixer, instead of the inlet pressure is proposed as the design variables, which is one of the novelties of the present work. The flow conservation at junction 10, 7, 8, and 9, is written as9
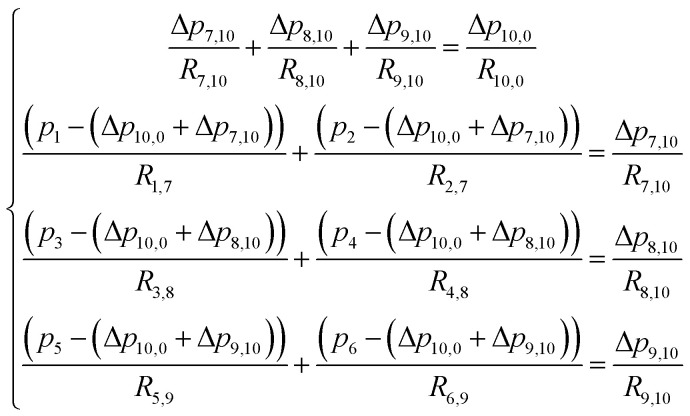
where as shown in Fig. 3, *p*, Δ*p*, and *R* are the pressure, pressure difference, and resistance, respectively; the subscript with a single number or two numbers, respectively, denote the quantity at the junction or the quantity across the channel, *e.g.*, pressure difference and resistance between two junctions. The pressure at the outlet is assumed zero in eqn (9), *i.e.*, grounded, and the pressure at junction 10, *i.e.*, *p*_10_ is equal to Δ*p*_10,0_. Therefore, the inlet pressures *p*_1_, *p*_2_, …, *p*_6_ can be expressed using pressure differences Δ*p*_7,10_, Δ*p*_8,10_, and Δ*p*_9,10_. In our formulation, the incoming branch channels of each Y-shaped mixer and the pressures at their inlets are set the same, while the pressures could be different from one Y-shaped mixer to another, that is, *p*_1_ = *p*_2_, *p*_3_ = *p*_4_, *p*_5_ = *p*_6_, and *p*_1_ ≠ *p*_3_ ≠ *p*_5_. By simply constraining the values of these pressure differences to be larger than zero, the backflow can be effectively eliminated because positive pressure differences of junction 7, 8, and 9 relative to junction 10 imply that all fluid streams enter the main output channel.

Mathematically, the SBO-based design of μCGGs can be summarized as follows:10
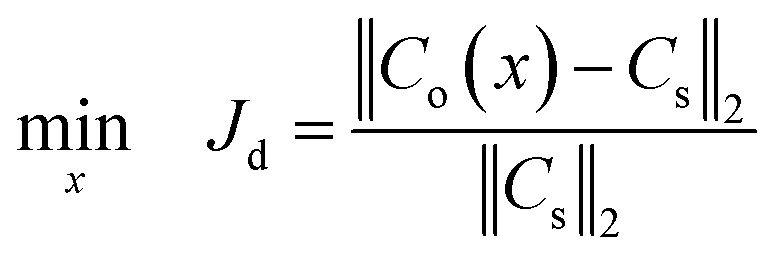
where *x* is the vector of the design variables; recall that *C*_s_ and *C*_o_ are, respectively, the prescribed CG and the CG of candidate design extracted at the detector location; and *C*_o_ depends on the values of the design variables *x*. The CG is measured with 100 uniformly distributed probes along the channel width direction, and thus, both *C*_s_ and *C*_o_ are a 100-dimensional vector.

**Fig. 4:**
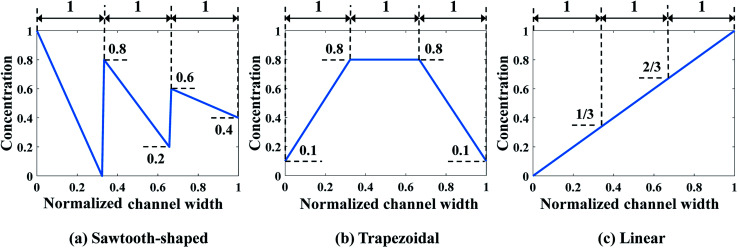
Prescribed CGs in the first case study: design of inlet concentrations.

In this paper, two case studies following the formulation above are investigated to verify SBO-based design of μCGGs. In the first one, only normalized chemical concentrations at the six inlets are included as the design variables, *i.e.*, *x* = [*c*_1_, *c*_2_, …, *c*_6_] with *c*_*i*_ being a scalar, and the pressure difference applied across the merging channel of all the Y-shaped mixers is the same, *i.e.*, Δ*p*_7,10_ = Δ*p*_8,10_ = Δ*p*_9,10_. This reduces the problem to six dimensions and is called design of inlet concentrations hereafter. In this case study, *p*_1_ = *p*_2_ = *p*_3_ = *p*_4_ = *p*_5_ = *p*_6_ = 382.44 pa is used, and correspondingly the flow rate through each inlet channel is fixed as 864 nl min^−1^.^12^Fig. 4 illustrates three prescribed CGs, *i.e.*, *C*_s_ that need to be generated by selecting appropriate inlet concentrations *c*_*i*_, which include the sawtooth-shaped, trapezoidal, and linear CGs. The normalized concentration is in the range of [0–1]. Note that due to the same flow rate through each inlet channel, three linear segments in the prescribed CGs have the same width.

**Fig. 5:**
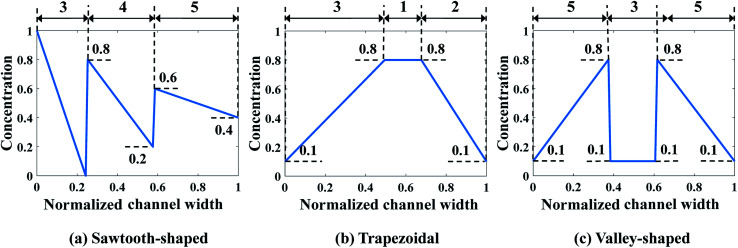
Prescribed CGs in the second case study: design of both inlet concentrations and pressure differences.

In the second case study, the pressure difference across the merging channel of the three Y-shaped mixers (Δ*p*_7,10_, Δ*p*_8,10_, and Δ*p*_9,10_ in Fig. 3), are introduced as three additional design variables to generate more complex CGs. This will increase the design dimension to 9, *viz*., *x* = [*c*_1_, …, *c*_6_, Δ*p*_7,10_, Δ*p*_8,10_, Δ*p*_9,10_], making it more challenging to construct the surrogate model and to search optimal parameters corresponding to minimum *J*_d_. Note that once the optimal values of the pressure difference are found, they can be converted to the inlet pressures at the reservoirs using eqn (9). Because of the identical size of the inlet branch channels in all Y-shaped mixers, the two inlet pressures and the two flow rates through each Y-shaped mixer are equal, that is *q*_1_ = *q*_2_, *q*_3_ = *q*_4_, and *q*_5_ = *q*_6_. However, the flow rate can be different among them, *i.e.*, *q*_1_ ≠ *q*_3_ ≠ *q*_5._ Therefore, the μCGG in this design can generate CGs comprised of three segments with different widths as shown in Fig. 5, which include the sawtooth-shaped, trapezoidal, and valley-shaped CGs.

## 4 Results and discussion

In this section, we will first describe a process to verify the optimum design obtained by SBO with adaptive sampling. Then the details of the SBO design solutions for both case studies above will be presented. Specifically, in each case study, the model selection step is first undertaken to compare various combinations of the regression model and the correlation model and select the best one for surrogate model construction. The adaptive sampling is then carried out to update the surrogate model and the response surface will be refined with infills for enhanced approximation, in which various infill criteria are also compared in terms of convergence rate. The performance of SBO with adaptive sampling will also be benchmarked with other relevant optimization methods, including SBO with random sampling and gradient-based optimization.

**Fig. 6:**
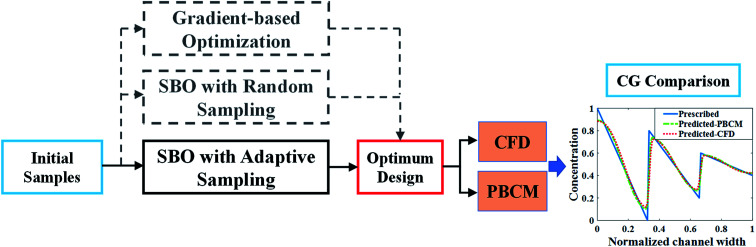
The procedure for design verification and performance benchmarking.

The procedure to verify the design obtained by SBO with adaptive sampling and benchmark its performance with the other optimization methods is illustrated in Fig. 6. Starting with a minimum number of initial samples, SBO with adaptive sampling (within the black box) consisting of model selection and infill will be performed, and eventually yields an optimum design when convergence criterion is reached. For verification, the optimum design parameters are then entered to CFD simulation or PBCM simulation, producing a CG that is then compared against the prescribed CG, *i.e.*, *C*_s_. The process will be repeated for several prescribed CGs as presented in Section 3. To quantitatively characterize the performance of the proposed design method, two performance criteria are defined, including discrepancy of PBCM *J*^PBCM^_d_ and discrepancy of CFD *J*^CFD^_d_11
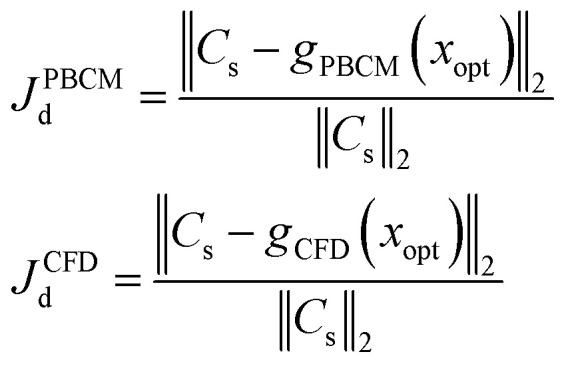
where *C*_s_ again is the prescribed CG; *x*_opt_ is the optimum design parameters; *g*_PBCM_ and *g*_CFD_ represent PBCM and CFD simulation, respectively, which takes *x*_opt_ as inputs and predicts the generated CGs. In this paper, CGs produced by CFD simulation is treated as the ground truth. *J*^PBCM^_d_ and *J*^CFD^_d_ are used to inspect different aspects of the design process. *J*^PBCM^_d_ compares the prescribed CG and the CG computed by PBCM using optimum design parameters, and therefore, it characterizes not only design performance, but also feasibility of generating the prescribed CG. It should be noted that it is almost impossible to generate prescribed CGs in Fig. 4 and 5 exactly using μCGGs due to the physical limitation that CGs will be bent at all channel walls due to their impermeability to species transport.^12^ More broadly, *J*^CFD^_d_ will also examine the discrepancy between PBCM and high-fidelity CFD arising from the assumptions used in PBCM. CFD simulation is performed with the commercial finite volume method (FVM) package CFD-ACE+ (http://www.esi-cfd.com) to verify the optimal parameters and corresponding CGs obtained by various design methods above. The details regarding CFD simulation is presented in our prior work.^12^

As discussed above SBO with adaptive sampling is also compared with two other design methods, *i.e.*, SBO with random sampling and gradient-based optimization as shown by the gray dashed lines in Fig. 6. In the former, the one-shot random sampling is used, and the surrogate model is only constructed once before the design process using simulation data at these randomly sampled parameters. In the latter, Matlab's built-in function, *fmincon*, a gradient-based optimization method to find the minimum of a constrained nonlinear multivariable function, is used to search for the optimum given prescribed CGs. The design performance of these methods, including accuracy and the numbers of evaluations, *i.e.*, design costs are also compared.

### 4.1 Case study 1: design of inlet concentrations

For each prescribed CG given in Fig. 4, a comparative analysis is performed to compare the performance of various combinations of the regression model and the correlation model in order to construct a surrogate model of salient accuracy for SBO design. 28 training data, the minimum number of required samples to build a surrogate model with the second order polynomial regression in the 6-dimensional design space is adopted.^21^ For each prescribed CG, a total of 15 surrogate models are constructed by the full-factorial combination of three regression models and five correlation models. The regression models under consideration include the 0^th^, 1^st^, and 2^nd^ order polynomial. The correlation models include spline, Gauss, exponential, linear, and spherical, and their mathematical expressions are described in ref. 23. Then surrogate model-predicted values are compared with true values of 9 validation data, and the relative error between them is defined as12
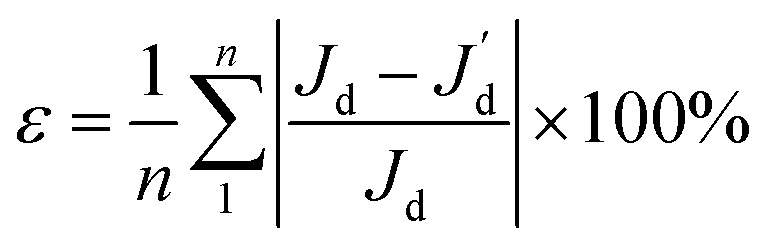
where *J*_d_ is the true value of the discrepancy between the prescribed CG *C*_s_ and the CG generated at the validation samples *C*_o_, while 
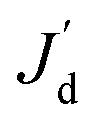
 is the discrepancy predicted by the surrogate model, *n* is the number of validation samples and is 9 in this case study. Table 1 lists the relative error for various combinations of the regression model and the correlation model, according to which the most accurate one is selected for adaptive sampling and SBO.

**Table 1:** Relative percentage errors of surrogate models built by different combinations of regression and correlation models in the case study: design of inlet concentrations

Regression model	Correlation model	Relative (percentage) errors *ε*		
		Sawtooth-shaped	Trapezoidal	Linear
Zero order polynomial	Spline	20.60%	22.56%	20.69%
	Gauss	17.63%	17.45%	12.78%
	Exp.	19.59%	21.18%	13.07%
	Linear	20.60%	22.56%	20.69%
	Spherical	20.60%	22.56%	20.69%
First order polynomial	Spline	15.20%	10.52%	10.10%
	Gauss	13.25%	9.48%	8.89%
	Exp.	15.20%	10.31%	10.59%
	Linear	15.20%	10.52%	10.10%
	Spherical	15.20%	10.52%	10.10%
Second order polynomial	Spline	16.14%	26.08%	9.60%
	Gauss	16.14%	26.08%	9.60%
	Exp.	16.14%	26.08%	9.60%
	Linear	16.14%	26.08%	9.60%
	Spherical	16.14%	26.08%	9.60%

**Fig. 7:**
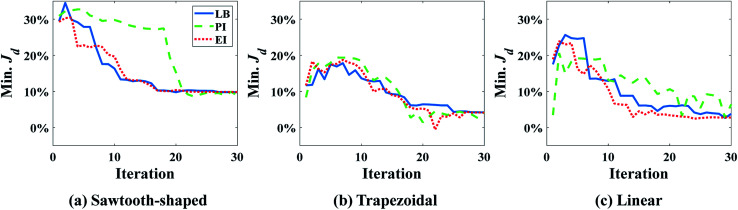
Convergence of Min. *J*_d_ using different infill strategies for prescribed CGs: (a) sawtooth-shaped, (b) trapezoidal, and (c) linear in the case study in the case study: design of inlet concentrations.

It was found that the first-order polynomial regression model combined with the Gauss correlation model reveals the smallest relative error *ε* for all three prescribed CGs, and thus, is selected for SBO with adaptive sampling. In this case study, 30 adaptive samples/infill (corresponding to 58 in total) are allowed to find the optimum design for each prescribed CG. For comparison, three infill techniques above are applied separately. Fig. 7 shows the convergence of Min. *J*_d_ of the surrogate model for different infill strategies for each prescribed CG. Multiple runs of the same optimization configuration are repeated for each prescribed CG, and all converge to the global optimum, and therefore, the same results are not duplicated here for the sake of conciseness. For sawtooth-shaped CGs, LB exhibits a faster convergence rate compared to the other two infill strategies. For the trapezoidal CG, three infill strategies have a similar convergence rate. For the linear CG, EI converges to a better solution, *i.e.*, lower *J*_d_ at a faster rate.

Table 2 lists the numerical values of *J*_d_ of optimum design in all cases above. The *J*_d_ achieved for each infill strategy represents the closeness between the CG generated by the candidate design and the prescribed CG. A better infill strategy constructs a more accurate surrogate model near the region around the optimum to capture the true response surface of *J*_d_ there, but not necessarily the entire design space. This is because the goal of our infill is to improve the accuracy of the optimum design, rather than building a global surrogate model to represent the input and output relationship across the entire domain. The results in Table 2 confirm that LB outperforms the other two for the sawtooth-shaped CG, all the three are equivalent for the trapezoidal CG and LB is used for the analysis below, and EI is the best the linear CG.

**Table 2:** Comparison of different infill strategies and prescribed CGs in terms of *J*_d_ of optimum design for the case study: design of inlet concentrations

Infill	*J*_d_		
	Sawtooth-shaped	Trapezoidal	Linear
LB	9.83%	4.07%	2.97%
PI	10.24%	4.54%	7.04%
EI	9.91%	4.20%	2.60%

**Fig. 8:**
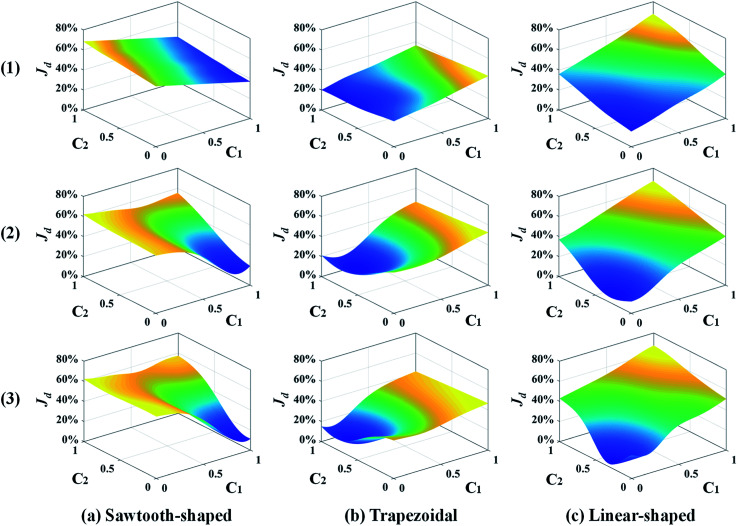
Response surface plots of the surrogate models in 3D that vary with *c*_1_ and *c*_2_ while keeping the other design variables constant for different numbers of sample infills: (1) 0, (2) 15, and (3) 30 in the case study: design of inlet concentrations.

Fig. 8 shows the response surfaces of *J*_d_ predicted by the surrogate models as more samples are added by the best infill strategy identified above for each prescribed CG. To facilitate visualization, the surface is portrayed in 3D that only varies with *c*_1_ and *c*_2_ while keeping the other design variables constant. 0, 15, and 30 sample infills are employed in the surface plot from the top to the bottom, respectively. It clearly shows that for each prescribed CG, the minimum value of the surrogate model becomes smaller and converges to a single point as more infills are added. Besides, the infill points are mostly distributed within the region close to the minimum, and impact the response surface shape there, which improves the accuracy of the optimum design solution and confirms that adaptive sampling effectively accelerates the process of search.

**Fig. 9:**
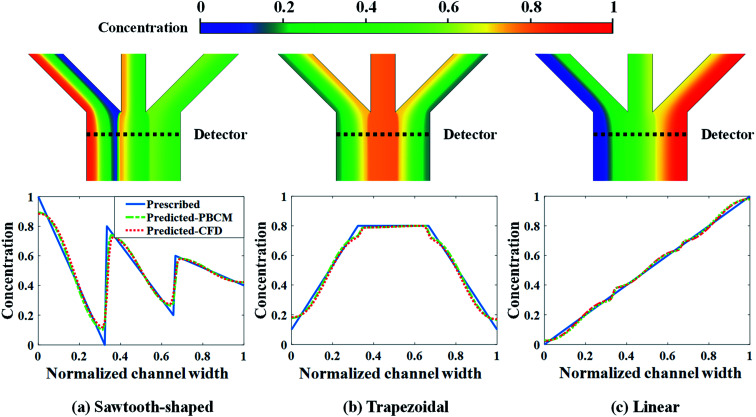
CFD contour plots and predicted CGs relative to the prescribed CG for the case study: design of inlet concentrations.

CFD simulation results and the comparison between the prescribed and predicted CGs in terms of the normalized chemical concentration are shown in Fig. 9. The concentration contour near the Ψ-shaped junction is displayed in the top row, and the CGs across the channel width are observed at the detector, which is located 400 μm downstream the Ψ-shaped junction. The PBCM- and CFD-predicted CGs match well with prescribed CGs, which are obtained by supplying the optimum designs to PBCM and CFD-ACE+ simulation. The excellent agreement of PBCM- and CFD-predicted CGs with respect to prescribed CGs verifies the accuracy of SBO with adaptive sampling for the μCGG design. However, minor differences are also observed at the stream interface and the side walls, which can be attributed to the fact that the prescribed CGs are created by concatenating three linear profiles while in actual μCGGs, CGs will be bent near all channel walls resulting from their impermeability to chemical species. In other words, the μCGG is not able to generate exactly the same prescribed CGs if the latter are artificial and do not fully match the solution of the underlying species transport equation. In addition, there is also an excellent match of the CG results predicted by PBCM and CFD, which implies that PBCM although with assumptions to allow analytical solution, is an accurate approximation of computationally demanding CFD, and can be used in place of the latter for design.

**Fig. 10:**
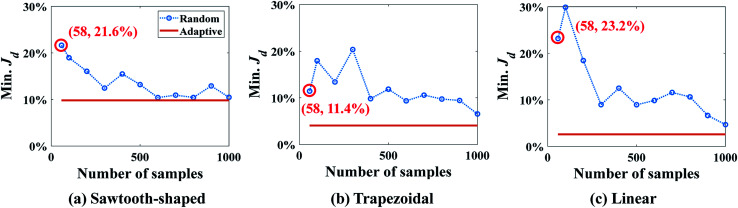
Comparison of results between SBO with random sampling and adaptive sampling for the case study: design of inlet concentrations.

The optimum design found by SBO with adaptive sampling is also compared with two other design methods, *i.e.*, SBO with random sampling and gradient-based optimization (enclosed in the dashed boxes in gray in Fig. 6), for all three prescribed CGs. In the former, one surrogate model is constructed using training data produced at parameters selected by one-shot random sampling before the design, and it is then used in the design process without infill or model update. Fig. 10 shows Min. *J*_d_ found by SBO with random sampling that uses the different number of samples, and compares it with the reference line in orange, *viz.*, adaptive sampling results using 58 samples in total. The red circle represents the result of random sampling when 58 samples in total are used. It clearly reveals that the optimal solution determined by the one-shot random sampling and corresponding surrogate model exhibit a much larger value of Min. *J*_d_ for all cases. The adaptive sampling at least improves the accuracy of random sampling by two times, that is, Min. *J*_d_ drops from 21.6% to 9.83% in the sawtooth-shaped CG, from 11.4% to 4.07% in the trapezoidal CG, and from 23.2% to 2.60% in the linear CG. Even if the number of randomly selected samples is increased to 1000, the accuracy of random sampling-based design cannot reach that by adaptive sampling. Besides, as we can see from the figures, the oscillation present in the curve of the random sampling is due to the insufficient number of samples. Therefore, given a fixed simulation budget, adaptive sampling is more computationally efficient and desired for global optimum search.

Next, SBO with adaptive sampling is compared with the gradient-based optimization method in terms of the total number of PBCM simulations, and the latter uses Matlab's built-in function, *fmincon*. It is well known that the number of model evaluations (*viz*., PBCM simulations herein) in gradient-based optimization heavily depends on the selection of initial start points. Therefore, ten runs with different initial points, which are selected from initial samples in SBO with adaptive sampling, are undertaken and examined for the gradient-based optimization. The number of PBCM evaluations required to reach the same accuracy as SBO with adaptive sampling in the ten runs is averaged, and the average number is then compared with that of the proposed method. Table 3 shows that for all three prescribed CGs, SBO with adaptive sampling uses a smaller number of PBCM evaluations/simulations (∼30 less on the average) for this case study involving 6 design variables.

**Table 3:** Comparison of the number of PBCM evaluation/simulation between SBO with adaptive sampling and gradient-based optimization for the case study: design of inlet concentrations

Method	Sawtooth-shaped	Trapezoidal	Linear
Gradient-based	100	87	109
SBO	58	58	58

### 4.2 Case study 2: design of inlet concentrations and pressure differences

Next, we extend the study to the 9-dimensional design space encompassing six inlet concentrations of chemicals and three pressure differences in all the Y-shaped mixers. Similarly, the best combination of the regression model and the correlation model is first selected through a comparative analysis. Each combination uses 55 samples in the training data, which is the minimum number of samples required to build a surrogate model with the second order polynomial regression in the 9-dimensional design space. Subsequently, 17 validation samples are utilized to evaluate performance of the 15 combinations of the regression model and the correlation model for all three prescribed CGs in Fig. 5. The relative percentage errors are listed in Table 4, which clearly indicates that the first-order polynomial regression model and the Gauss correlation model yields the smallest relative errors *ε* and is the best one for all three cases.

**Table 4:** Relative percentage errors of surrogate models built by different combinations of regression and correlation models in the case study: design of inlet concentrations and pressure differences

Regression model	Correlation model	Relative (percentage) error *ε*		
		Sawtooth-shaped	Trapezoidal	Valley-shaped
Zero order polynomial	Spline	17.87%	28.90%	18.54%
	Gauss	17.57%	25.21%	17.57%
	Exp.	17.87%	28.90%	18.54%
	Linear	17.87%	28.90%	18.54%
	Spherical	17.87%	28.90%	18.54%
First order polynomial	Spline	16.55%	20.46%	11.64%
	Gauss	16.48%	20.44%	11.10%
	Exp.	16.55%	20.46%	11.64%
	Linear	16.55%	20.46%	11.64%
	Spherical	16.55%	20.46%	11.64%
Second order polynomial	Spline	83.02%	56.99%	83.66%
	Gauss	83.02%	56.99%	83.66%
	Exp.	83.02%	56.99%	83.66%
	Linear	83.02%	56.99%	83.66%
	Spherical	83.02%	56.99%	83.66%

**Fig. 11:**
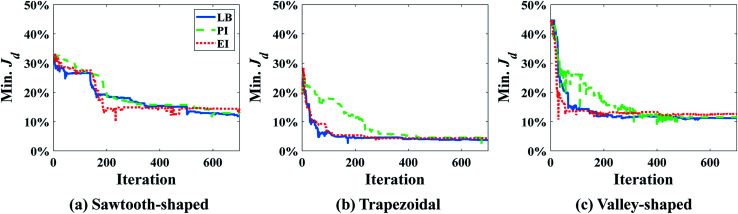
Convergence of Min. *J*_d_ of surrogate model using different infill strategies for prescribed CGs: (a) sawtooth-shaped, (b) trapezoidal, and (c) valley-shaped for the case study: design of inlet concentrations and pressure differences.

With the best surrogate model structure selected, SBO design with adaptive sampling is then carried out subject to a budget of 700 infill samples that are selected by three different infill strategies. Fig. 11 portrays convergence curves of Min. *J*_d_ of the surrogate model using the three infill strategies for each prescribed CG. LB outperforms the other two in terms of the convergence rate, and constructs more accurate surrogate models that find inlet concentrations and pressure differences. This is quantitatively confirmed by *J*_d_ of optimum design listed in Table 5, which clearly shows that LB achieves the lowest *J*_d_ for all three prescribed CGs, yielding better designs than PI and EI.

**Table 5:** Comparison of different infill strategies and prescribed CGs in terms of *J*_d_ of optimum design for the case study: design of inlet concentrations and pressure differences

Infill	*J*_d_		
	Sawtooth-shaped	Trapezoidal	Valley-shaped
LB	11.90%	3.75%	11.23%
PI	12.97%	4.36%	11.57%
EI	14.61%	4.43%	12.77%

**Fig. 12:**
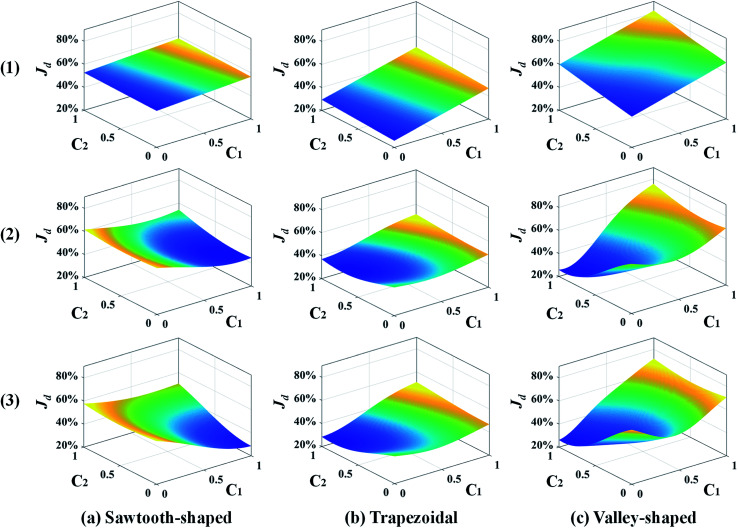
Response surface plots of the surrogate models in 3D that vary with *c*_1_ and *c*_2_ while keeping the other design variables constant for different numbers of sample infills: (1) 0, (2) 300, and (3) 700 in the case study: design of inlet concentrations and pressure differences.

The response surfaces of *J*_d_ predicted by the surrogate models with more samples added by the LB infill are shown in Fig. 12. Similarly, for the sake of visualization, only *c*_1_ and *c*_2_ vary while the other design variables are held constant. The surface plots from the top to the bottom are generated by surrogate models with 0, 300, and 700 infills. It shows that without infills, the response surfaces appear almost linear because of the use of the first-order regression model and extremely insufficient training data. As more infill points are added, *e.g.*, 300 infills, the nonlinearity of the response surface for each prescribed CG is observed, and Min. *J*_d_ becomes evident. The profiles of the response surfaces only change slightly at 700 infills along with the converged solution of Min. *J*_d_. Again, this confirms that the surrogate model can be improved and the optimum design in 9-dimensional space can be found in a reliable manner given adequate infill points. More importantly, adaptive sampling based on the LB infill strategy successfully assigns most of the infill points within the region close to optimum (not shown to avoid data clustering and facilitate visualization) that provides more topological information to speed up the search process and improve the solution accuracy.

**Fig. 13:**
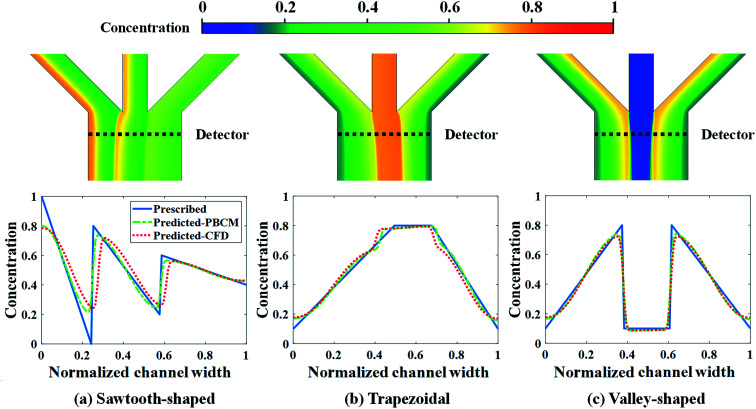
CFD contour plots and predicted CGs relative to the prescribed CG for the case study: design of inlet concentrations and pressure differences.

**Fig. 14:**
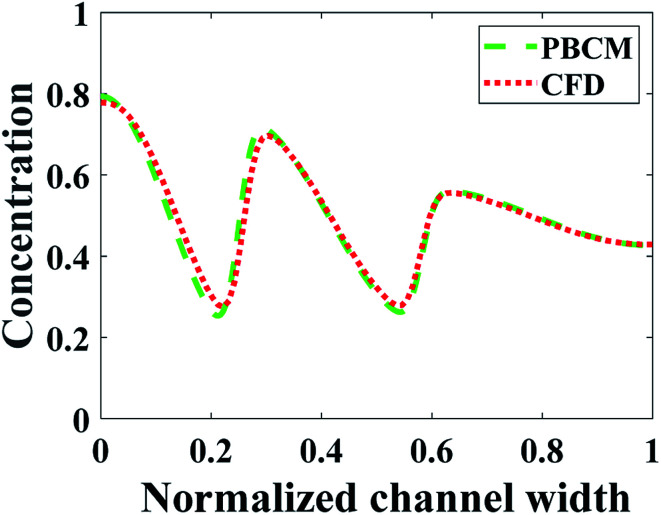
Comparison between PBCM and CFD simulation at fully developed region for the sawtooth-shaped CG.

Fig. 13 shows the CFD contour plots of the normalized chemical concentrations and the comparison between the prescribed and predicted CGs extracted at the detector location. Table 6 lists the numerical values of *J*_d_s of CGs predicted by PBCM and CFD simulation using the optimum design parameters found above by SBO with adaptive sampling. There are several points to note. First, the PBCM-predicted CGs match well with prescribed CGs in all cases, although their shapes are more complex in this case study with 9 design variables. The discrepancies between prescribed CGs and PBCM-predicted CGs are mostly due to bending of the concentration distributions at the side walls that are impermeable to chemical transport. Second, CFD-predicted CGs exhibit noticeable discrepancy from the prescribed and PBCM-predicted CGs in the sawtooth-shaped and trapezoidal CGs, and therefore, the values of *J*_d_ by CFD is appreciably higher than that of PBCM in both CGs. This is caused by appreciable transverse flow immediately downstream the Ψ-shaped junction before flow is fully developed, and presence of the detector within the flow entrance region of the main output channel, as revealed by the concentration contours in Fig. 13a and b. However, as discussed above, our PBCM is not able to take into account such an effect because of its modeling assumptions. To confirm the interpretation, Fig. 14 illustrates the comparison between PBCM- and CFD-predicted CGs when the detector is located further downstream (2000 μm from the Ψ-shaped junction), and we can see that both match very well. It is also noticed that for the valley-shaped CG, the transverse flow is relatively weak due to the pressure symmetry in the streamwise direction, which again is apparent in CFD contour plot of Fig. 13c. Therefore, PBCM- and CFD-predicted CGs are almost identical with negligible differences.

**Table 6:** *J*_d_s of CGs predicted by PBCM and CFD using the optimum designs found by SBO with adaptive sampling

	Sawtooth-shaped	Trapezoidal	Valley-shaped
PBCM	11.90%	3.75%	11.23%
CFD	22.98%	6.42%	12.34%

**Fig. 15:**
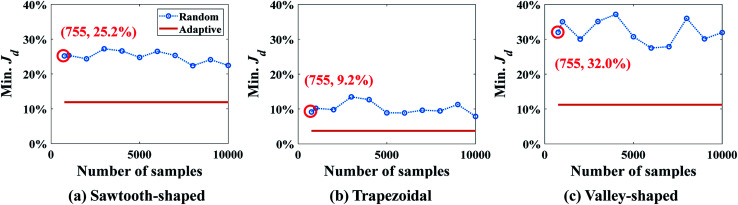
Results of SBO with random sampling for the design of inlet concentration and pressure difference.

Min. *J*_d_ for the different numbers of samples obtained by SBO with random sampling is shown in Fig. 15, and compared with shows the reference line in orange, *viz*., adaptive sampling results using 755 samples in total. Again, Min. *J*_d_ achieved by the random sampling is at least 2 times larger than that by adaptive sampling with 755 samples in total. Even if the number of samples and evaluations is 13 times larger than adaptive sampling, it still cannot reach the same level of accuracy. This is because even 10 000 samples for 9 dimensions are still too small for constructing an accurate surrogate model in the entire design space, in particular, around the region of optimum. It turns out that adaptive sampling effectively accelerates the process of searching minimum. Besides, the uniform distribution of samples contributes to the oscillation of the curve as the correlation matrix in Kriging can vary dramatically and randomly. However, the general trend of random sampling error is to decrease as more samples are added.

Table 7 shows the number of evaluations required by the gradient-based optimization to reach the optimum solution at the same level of accuracy as SBO with adaptive sampling. Again, 10 different start points are selected from the initial samples used in SBO design to initiate 10 gradient-based optimization runs. It shows that 50% of the runs fail to converge to the optimum solution for the sawtooth-shaped CG, 10% failure for the trapezoidal, and 50% for the valley-shaped. It seems caused by being trapped at local optima because Min. *J*_d_s achieved in these failed runs are notably higher than those at the global optimum. This implies that although gradient-based optimization can be more computationally efficient for high-dimensional design problems, there is a risk of missing the global optimum, in particular, for generating complex CGs. However, for the trapezoidal CG, gradient-based optimization outperforms SBO with adaptive sampling in efficiency for 9 runs, which may be attributed to the simpler topology of its response surface. Generally speaking, because of its exploratory nature, SBO with adaptive sampling is a more feasible and reliable method to search for the global optimum, while the gradient-based approach is more computationally efficient and requires fewer PBCM evaluation in high-dimensional design space if not trapped at the local optimum. The large variation in the number of PBCM evaluations further reveals that the gradient-based optimization is indeed dependent on the initial start point.

**Table 7:** Evaluation comparisons between the SBO with adaptive sampling and the gradient-based optimization methods

Run no.	Sawtooth-shaped		Trapezoidal		Valley-shaped	
	Number of evaluation	Min. *J*_d_	Number of evaluation	Min. *J*_d_	Number of evaluation	Min. *J*_d_
1	247	11.42%	448	3.74%	Fail	22.37%
2	280	11.17%	207	3.73%	Fail	22.36%
3	Fail	31.11%	246	3.70%	307	10.83%
4	484	11.12%	255	3.69%	260	10.93%
5	Fail	31.11%	204	3.75%	Fail	21.99%
6	279	10.63%	195	3.72%	229	11.18%
7	297	11.06%	219	3.74%	Fail	21.93%
8	Fail	31.11%	195	3.73%	255	11.15%
9	Fail	31.11%	216	3.71%	351	10.96%
10	Fail	31.11%	Fail	4.62%	Fail	22.28%

## 5 Conclusion

In this paper, a new method based on surrogate-based optimization (SBO) with adaptive sampling is developed for efficient and reliable design of microfluidic concentration gradient generators (μCGGs). The key rationale of the proposed method is to construct the surrogate model, *i.e.*, Kriging, using physics-based simulation data, update the model using incrementally and adaptively added data, *viz*., infill, and then utilize continuously enriched topological information provided by the surrogate model to guide the search of global optimum. New aspects of the proposed research include: first, the feasibility of applying SBO with adaptive sampling to complex μCGG design is systematically examined. Second, the physics-based component model (PBCM) of μCGGs in the closed-form is employed to generate data for surrogate model construction to further reduce the computational cost. Third, a comparative analysis is performed to identify the best combinations of the regression model and the correlation model, and determine the infill strategies for improved surrogate modeling and design performance. Last, the use of pressure differences rather than the native inlet pressures as the design variables to eliminate the backflow issue.

Two case studies are undertaken on the partial mixing- and species transport-governed triple Y-shaped μCGG to evaluate design performance of the proposed method. Key technical findings are obtained, including

(1) Our comparative analysis indicates that combining the first-order polynomial regression model and the Gauss correlation model in Kriging yields the highest surrogate model accuracy.

(2) In general, three infill strategies all allow the design to converge to the global optimum, while on average LB exhibits faster convergence rate than EI and PI.

(3) All CGs predicted by PBCM using the optimum design parameters match prescribed CGs, which verifies feasibility, robustness, and accuracy of the proposed method.

(4) Both PBCM-predicted and CFD-predicted CGs match very well in the first case study, which validates the accurate design of SBO with adaptive sampling, while an appreciable difference (average *J*_d_ difference 4.95%) between them is observed in the second case study. It is attributed to asymmetric flow rates through Y-shaped mixers in the second case study that give rise to significant transverse flow in the entrance region, the effect of which on species transport can be captured by CFD but not PBCM.

(5) The proposed method is at least two times more accurate than SBO with random sampling for all prescribed CGs, which confirms that adaptive sampling-based infill is necessary for SBO design of complex CGs.

(6) The gradient-based optimization method requires at least 30 more evaluations compared to the method of SBO with adaptive sampling in the first case study. In the second case, approximately 1/3 of the runs of gradient-based optimization fail to find the global optimal solution, although on average it uses less simulation than SBO with adaptive sampling. In short, SBO with adaptive sampling is preferred as a robust method to find the global optimum design.

The future work will focus on combining a small amount of high-fidelity CFD data with PBCM data to construct multi-fidelity surrogate models^37^ to address the discrepancy between PBCM- and CFD-predicted CGs caused by transverse flow in the entrance region, and utilize the multi-fidelity model for μCGG design.

## Conflicts of interest

There are no conflicts to declare.

## Supplementary Material
